# Exploring the Relationship between Despotic Leadership and Job Satisfaction: The Role of Self Efficacy and Leader–Member Exchange

**DOI:** 10.3390/ijerph18105307

**Published:** 2021-05-17

**Authors:** Xiang Zhou, Samma Faiz Rasool, Jing Yang, Muhammad Zaheer Asghar

**Affiliations:** 1 School of Management, Guangzhou University, Guangzhou 510006, China; zx@gzhu.edu.cn (X.Z.); yangj68@chinatelecom.cn (J.Y.); 2 Postdoctoral Station of Statistical, Guangzhou University, Guangzhou 510006, China; 3 School of Innovation and Entrepreneurship, Entrepreneurship Institute, Guangzhou University, Guangzhou 510006, China; 4 Education & ICT (e-Learning), Universitat Oberta de Catalunya, 08018 Barcelona, Spain; zaheer.asghar@helsinki.fi; 5 Faculty of Educational Sciences, University of Helsinki, 00014 Helsinki, Finland

**Keywords:** despotic leadership, job satisfaction, self-efficacy, leader–member exchange, small- and medium-size enterprises (SMEs)

## Abstract

This study explores the effects of despotic leadership (DL) on employee job satisfaction (JS) using self-efficacy (SE) as a mediating variable and leader–member exchange (LMX) as a moderated variable. Building on the social learning and social exchange theory, our research proposes a research model. In this model, despotic leadership affects employee job satisfaction both directly and indirectly through self-efficacy and leader–member exchange. We used a questionnaire survey analysis approach to collect data. Data were collected from the employees of small- and medium-sized enterprises (SMEs) located in Guangdong Province, P.R. China. A pilot test of 20 participants with similar demographics as the final sample was performed to test the usability of the questionnaire. We distributed 500 questionnaires among the target population. In total, 230 usable questionnaires were returned, resulting in a response rate of 53%. To estimate the proposed relationships in the theoretical framework, we used SPSS and AMOS. The results of this study confirmed that despotic leadership has a negative impact on employee job satisfaction. Moreover, the outcomes of this study indicate that self-efficacy has a mediating effect between despotic leadership and employee job satisfaction. Similarly, the results also confirm that LMX has a moderating effect between despotic leadership and employee self-efficacy. Therefore, we conclude that the community is understanding of the mechanism of despotic leadership, identify the mechanism to effectively deal with its negative effects, broaden the relevant research on the antecedent variable of self-efficacy, and provide practical enlightenment enterprises to retain and employ people.

## 1. Introduction

China has experienced dramatic industrialization and economic development over the last three decades. Therefore, over the last three decades, small- and medium-sized enterprises (SMEs) have played an essential role in industrialization and economic development because they provide about eighty percent of jobs in China [1]. However, compared to the corporate sector, small- and medium-sized enterprises generally have low wages and a high level of toxic workplace environments because of despotic leadership [2]. Since low wages and a toxic workplace environment demotivate the employees, which affects their job satisfaction, many prior studies have found that despotic leadership in SMEs negatively impacts employees’ job satisfaction [3,4,5]. 

In the era of the knowledge economy, whether or not to create a working environment that stimulates employee potential is a key factor for whether enterprises remain competitive [6,7]. Moreover, in Eastern countries where the “rule by man” culture prevails, leadership style, as an important working environment factor, has a particularly prominent influence on employees [8]. On the positive side, charismatic leaders are like a fountain of sunshine to employees. Conversely, despotic leadership can lead employees towards destruction [9,10]. Thus, in terms of talent motivation, “the success or failure of the affair is all due to the leadership.”

The relevant research on leadership covers a broad discussion on the positive influence of leadership on employee motivation [11]. According to these studies, leadership has a significant impact on employees’ long-term career growth and development, as well as their current work, psychological state, and behavioral performance [12]. Furthermore, numerous scholars have also performed rich explorations of the positive effects of leadership style (such as transformational, intelligent, and charismatic leadership) on employees. They confirm that leadership style plays a positive role in promoting employee creativity (Herrmann and Felfe [13]), forming a sense of belonging and organizational commitment Top, Akdere [14] and improving their work efficiency [15]. However, due to the influence of traditional culture, employees in Eastern countries tend to adopt a tolerant and silent attitude towards despotic leadership behaviors such as control and suppression. Therefore, relevant investigations on leadership have been in a state of “reporting good news but not bad news” for a long time. Therefore, how does a negative leadership style affect employees’ job satisfaction? There is still a lack of in-depth discussion in the existing literature. 

With the spread of information and culture change, employees increasingly affected by negative leadership styles are no longer silent. They bravely express their dissatisfaction with “bad leaders” by leaving their jobs or exposing their leaders. Driven by these phenomena, the dark and destructive sides of leadership behavior have gradually attracted the attention of relevant scholars [16,17]. Given the significant impact of destructive leadership behavior on corporate culture, organizations, and individuals, and the fact that relevant data have become increasingly accessible, the relevant empirical studies show an increasing trend [18]. Despotic leadership, which emphasizes the absolute authority of the leader and requires the absolute obedience of subordinates, is a representative type among all destructive leadership behaviors [19]. Moreover, this type of leadership is ubiquitous in business organizations in the Oriental and Asia-Pacific cultural contexts, and thus, it is of great research value [8]. However, due to its negative effects on leadership, despotic leadership research is particularly limited, and the recent findings in the literature are insufficient to provide a comprehensive and in-depth understanding of the specific path through which it affects employee job satisfaction and the prevention mechanism of related negative effects [19]. Moreover, the current literature cannot provide companies and employees with effective guidance and inspiration in dealing with the negative effects of despotic leadership.

There are some gaps in the current literature regarding providing guidance and inspiration for employees to cope with the negative effects of despotic leadership [8]. Despite the strong interest among researchers and practitioners, some gaps remain in establishing the interrelationship of despotic leadership and job satisfaction. First, most of the research focused on the positive side of leadership, and few researchers were concentrated on the negative effects of despotic leadership. In particular, this is the first study in which we test the negative aspects of despotic leadership and its relationship with job satisfaction, particularly in emerging countries such as China. Second, the despotic leadership practices in SME organizations are not a major focus of the literature. Third, few studies have examined the direct relationship between despotic leadership and job satisfaction or between despotic leadership and leader–member exchange, but the four-way relationship between despotic leadership, leader–member exchange, self-efficacy, and job satisfaction is unexplored. In particular, studies have not considered self-efficacy as an intervening construct or leader–member exchange as a moderating construct between despotic leadership and job satisfaction.

Based on the abovementioned research impetus, the purpose of this study is to analyze the gaps between the relationships of despotic leadership, leader–member exchange, self-efficacy, and job satisfaction. On the basis of the above discussion, the following three research questions are addressed:

RQ1: Does despotic leadership harm the employees’ job satisfaction?
RQ2: How does leader–member exchange moderate the relationship between despotic leadership and self-efficacy?
RQ3: How does self-efficacy intervene between despotic leadership and employee job satisfaction?

The paper is structured as follows: the second part presents the literature about despotic leadership, leader–member exchange, self-efficacy, and job satisfaction. The third section presents the hypothesis development, and the fourth part of this study is about the research methodology. The fifth part of this study presents the discussion. The last section presents the conclusion and limitations. 

## 2. Literature Review

### 2.1. Despotic Leadership (DL)

Despotic leadership originated from the research on Taiwanese enterprises in China in the 1970s and is considered an important part of patriarchal leadership [20]. As an independent leadership style, such leadership has attracted wide attention from management circles, and has been studied by scholars all over the world [21]. Influenced by the Confucian value system, the father has absolute authority over family members, especially his children, in traditional Chinese families [22]. In more traditional Chinese enterprises, leaders usually choose to act as the father in an extreme leadership style to establish a centralized hierarchy that is easy to manage, so despotic leadership is prevalent in Chinese organizations [23]. Despotic leadership emphasizes absolute control over employees and is an ubiquitous leadership style in the modern society of collectivism and high efficiency. Despotic leadership is conceptualized as a leadership behavior in which leaders advocate supreme severity and absolute domination over subordinates and require them to obey unconditionally [24]. 

Farh and Cheng [20] describe despotic leadership as having four typical manifestations. First, the leaders have rigorous control over their subordinates, and such leaders want their subordinates to obey them. Second, despotic leaders are not accepting of any idea or suggestion from their subordinates. Such kinds of leaders take credit for successes and place the blame for failures on their subordinates. Third, despotic leaders usually seem very confident, and are sensitive to whether others respect them enough. Such kinds of leaders manipulate information and take advantage of others. Fourth, despotic leaders are rigorous, even harsh, with their subordinates. They are almost never satisfied with the work of their subordinates. 

### 2.2. Job Satisfaction (JS) 

As one of the most important organizational behavior concepts, job satisfaction is a significant psychological indicator of business management [25,26]. Since the birth of management science, job satisfaction has become a significant concern for international scholars. During its evolution, the concept of job satisfaction has embraced many different definition categories, such as comprehensiveness, difference, and reference structure [27]. Although disputes remain in the academic circle in relation to whether job satisfaction should be the subjective response or objective evaluation of employees in work situations, both alternatives are quite reasonable from the perspective of existing practices [28]. Therefore, from the perspective of data availability, this study defined job satisfaction as the sample’s emotional response to their work, namely, their subjective views and attitudes toward their work content, results, and rewards.

### 2.3. Self-Efficacy (SE)

Self-efficacy is a personal judgment of how well or poorly a person can cope with a given situation based on their skills and the circumstances they face [29]. Similarly, self-efficacy refers to how confident people feel that they can use the skills that they possess to perform certain tasks [30]. Bandura [31] believes that there is also an expectation of efficacy, in addition to the expectation of results. Outcome expectation is a person’s assumption that a certain behavior will lead to a certain outcome. If a person anticipates that a particular behavior will lead to a particular outcome, the behavior may be activated and selected [32]. Many factors influence the formation of self-efficacy. Four factors attract the most attention in academic circles, including the success or failure experienced by the research subjects, vicarious experiences, verbal persuasion of others, and physiological and emotional state [33]. A strong sense of self-efficacy promotes human accomplishment and personal well-being [34]. A person with high self-efficacy views challenges as things that are supposed to be mastered, rather than threats to avoid. These people can recover from failure faster and are more likely to attribute failure to a lack of effort [35]. They approach threatening situations with the belief that they can control them. These things have been linked to lower levels of stress and a lower vulnerability to depression [36].

### 2.4. Leader-Member Exchange (LMX)

The construct of leader–member exchange consists of the differences in the degree of closeness between leaders and their employees. Leaders treat employees differently due to their contributions, personal preferences, etc., which then gradually evolves into leader–member exchange relationships with different qualities [37]. A high-quality leader–member exchange relationship leads leaders to regard employees as “insiders”, while employees in low-quality exchange relationships are regarded as “outsiders” [38]. There are more emotional connections between the insiders and the leaders, and they are more trusted and cared about by the leaders. Additionally, insiders are more active when obeying the leaders, and they can give full play to their intelligence to finish the tasks. However, the relationships between the outsiders and the leader are formed on the basis of structural power, which is a purely working relationship. They have little contact with the leader and rarely get extra rewards or opportunities from the leader [39]. 

## 3. Hypotheses Development

### 3.1. Despotic Leadership and Employee Job Satisfaction

According to Herzberg [40], the factors that affect job satisfaction can be divided into two categories: the employees themselves and the working environment [40]. In recent years, an increasing number of studies have found that leadership style, as one of the key environmental factors, has a significant impact on employee job satisfaction [41]. Among those scholars, Top and Akdere [14] demonstrate that transformational leadership can effectively improve employee job satisfaction, and, thus, facilitate the generation of organizational commitment and trust [42]. Braun and Kark [43] also found that transformational leadership can optimize employees’ production performance by improving job satisfaction [42,44]. Moreover, from an ethical leadership standpoint, some scholars also explored the impact of appropriate leadership behaviors on employee job satisfaction [45]. According to the existing research on leadership and job satisfaction, both transformational leadership and ethical leadership are relatively positive and can generate good work experience. Empirical studies on the mechanism by which a negative leadership style affects employee job satisfaction are relatively rare. To examine whether negative leadership style has an adverse effect on employee job satisfaction as opposed to a positive leadership style, this study selects despotic leadership, a representative negative leadership style, for in-depth analysis. This work argues that despotic leaders prefer self-centered behaviors such as dictatorial power, showing authority image, belittling employees’ working ability, and berating them, and these behaviors tend to make employees lose self-confidence and passion for work, thereby negatively impacting their job satisfaction [19,46]. Above all, this paper proposes that:

**Hypothesis** **1** **(H1).** *Despotic leadership has a negative impact on employee job satisfaction.*

### 3.2. Mediating Role of Self-Efficacy 

Social learning theory is an important scheme for exploring the process of human cognition formation [47]. According to this theory, driven by goals, individuals will form relevant expectations and action plans in combination with the evaluation of themselves and the environment, and then constantly reflect on themselves in the process of action to form the evaluation of self-efficacy, which will ultimately affect their expectations of themselves and their behavior. Self-efficacy refers to an individual’s belief that they can achieve a specific achievement [48]. Therefore, when a person has a high level of self-efficacy in a particular area, they are more likely to engage in related activities, and continued success leads them to be more interested in those activities [49].

Based on social learning theory, we posit that the control and intervention of despotic leaders will seriously affect employees’ control over their work, making them unable to gain the confidence that they can independently cope with challenges and control their destiny from work tasks, thereby making them unable to experience the value and significance of their work. Therefore, this work assumes that despotic leaders can weaken employees’ job satisfaction by lowering their self-efficacy. The specific logic is as follows:

First, self-efficacy refers to an individual’s speculation and judgment on whether they can complete a certain behavior [50]. According to social learning theory, people’s sense of self-efficacy often comes from their past social experiences [47]. For example, independently completing a certain task and achieving success can bring people a satisfactory experience and arouse their ambition to continuously participate and continuously improve this task [50]. In practice, employees’ sense of self-efficacy first comes from the experience of independently completing a task and achieving success. These experiences reinforce the employee’s perception that they are suitable for the job, and even have a talent for it. Furthermore, according to the achievement motivation theory, people always want to be successful and surpass others [51]. Thus, the inference can be made that continuing to do what you think you are good at is more likely to lead to more comfortable work experiences, higher achievement experiences, and material rewards.

Second, as the power center in the organization, leaders can control employees’ job opportunities and resources and directly determine their promotion opportunities through organization management and the assessment of their work performance [52,53]. Especially in the Oriental organization context with a strict “rule by man” climate, the degree of leadership intervention is higher in employees’ career development [8]. The reason for this intervention might be that despotic leaders like to control and master everything, believing that they should be in charge of every detail of the job. Moreover, those leaders like to take credit for the team’s work and blame the team’s mistakes on their employees [20]. By working under such a leader, employees will gradually lose self-efficacy for work, mainly for two reasons. On the one hand, employees will experience a lack of control over work tasks. Employees working under despotic leaders lack the necessary control and decision-making power for the work tasks that they are responsible for, and yet, often need to “bear the blame” when encountering problems [19]. Using the theory of social learning, one may readily infer that if employees lack decision-making power, then achievements are not recognized, but mistakes are punished; thus, the employees will develop a negative work attitude of “doing less is better than doing more”, and will no longer have the confidence to do a good job [32]. On the other hand, employees lack a deep understanding of their own abilities. According to the social learning theory, employees’ cognition of their own abilities is usually acquired while completing certain tasks independently and achieving success [39]. In this process, if the task is affected or interfered with by external forces, employees will encounter difficulty in clarifying the contribution of their input to the success or failure of the task. Therefore, it would be difficult for them to gain sufficient confidence from the relevant task experience. Above all, employee development expectations play an intermediary role between despotic leadership and employee job satisfaction. This inference is also in line with the prediction of social learning theory, and self-efficacy is an important source of motivation for most employees to engage in their work actively and confidently (Salazar and Hayward, 2018). Under the restrictions and constraints of despotic leadership, employees are unable to express their ambitions and upgrade their abilities and confidence by completing tasks independently. Consequently, they will gradually fall into the dilemma of “learned helplessness” and will naturally find it difficult to feel the value and joy brought about by work. Therefore, we propose:

**Hypothesis** **2** **(H2).** *Self-efficacy mediates between despotic leadership and employee job satisfaction.*

### 3.3. The Moderated Role of LMX

Under the “learned helplessness” brought about by despotic leadership, can employees with achievement motivation change this unfavorable situation through their own subjective initiative? This study tries to find solutions to this query from the perspective of social exchange theory. Accordingly, this work measures the degree of social exchange between leaders and employees with the concept of “leader–member exchange” [54]. The leader–member exchange (LMX) relationship refers to the quality of the relationship between leaders and subordinates as well as the degree of emotional support and resource exchange, and has been one of the hot topics in leadership theory in recent years [38]. Leader–member exchange theory holds that leaders and employees divide subordinates into two groups based on emotion, loyalty, perceived contribution, and professional respect in the exchange relationship between leaders and employees. One group consists of the “insiders”, loyalists who obey and follow their leaders. The other is comprised of “outsiders”, or the employees who are more detached from and do not fully follow the leadership [55]. Under the governance of a despotic leadership, the treatment of “insiders” and “outsiders” can differ entirely.

First, despotic leaders’ desire for control often comes from a lack of trust. By becoming an “insider” of the leader, promoting the trust between the leader and employees is conducive to promoting the leadership’s empowerment. Although despotic leaders are autocratic and self-centered, they are often sensitive and suspicious and do not easily trust others [9]. According to social exchange theory, employees can enhance mutual understanding and promote mutual trust based on full social exchange [56]. Therefore, effective leader–member exchange can be postulated to promote the despotic leaders’ trust in their employees and increase their motivation to full empowerment.

Second, despotic leaders tend to regard employees as tools in utility-oriented relationships, and employees are more likely to be treated humanely by leaders in emotion-oriented relationships. According to the theory of social exchange, the leader–member exchange affects self-efficacy [56]. Moreover, social exchange theory recommends that every employee has different behaviors so that the leader can affect their subordinate’s behaviors that affect their self-efficacy [57]. For example, for strangers and weak relationships, employees usually follow utility-oriented exchange rules, in which they are more concerned about gains and losses and prefer to get rather than give. On the contrary, when facing strong relationships with a colleague, employees usually follow the principle of emotion-oriented exchange. Under this exchange rule, employees are relatively less concerned about gains and losses and are more willing to give. Therefore, this discussion has proven that despotic leadership and leader–member exchange affect self-efficacy [58].

**Figure 1:**
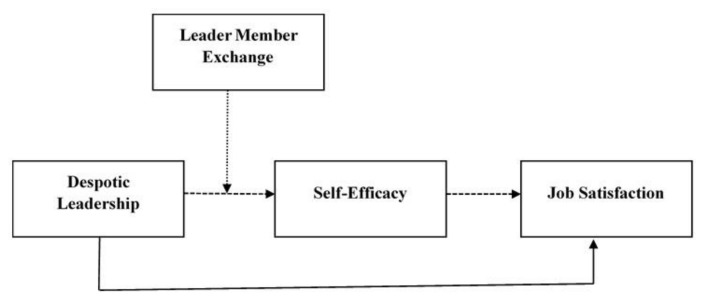
Research model: solid arrows (____) indicate a direct relationship, dashed arrows (_ _ _ _) indicate a mediation relationship, and dashed arrows (.....) indicate a moderating relationship.

Moreover, the relationship between individuals is not constant. Strangers and weak relationships may evolve into acquaintances and strong relationships through continuous social exchange [59]. Therefore, if the exchange of leaders and members can be strengthened and the exchange rules of despotic leaders for employees change from a utilitarian orientation to an emotional orientation, despotic leaders may also become willing to cultivate employees and grant them authorization and support for the purpose of cultivation. According to social learning theory, employees who are trusted, supported, and fully authorized by leaders are more likely to feel confident in their work and to gain a sense of self-efficacy from successful experiences [60]. As per our literature review, we have proposed the following hypothesis. Moreover, we have proposed a research model for this study (Figure 1).

**Hypothesis** **3** **(H3).** *Leader member exchange moderates between despotic leader ship and employee self-efficacy.*

## 4. Research Methods

### 4.1. Research Approach

In this research, we used a questionnaire survey analysis approach to collect data. We used this approach for three main reasons: First, the influence of leaders on employees is subtle and significant, so it is reasonable to measure employees’ feelings and feedback on leadership behaviors through self-reported questionnaires. Secondly, the analysis results of a large sample questionnaire can effectively test the scientific nature and universality of the research hypothesis. Finally, through the professional services provided by wjx.com and the researchers’ extensive contacts in the Guangdong region, China, we can easily access a large number of reliable samples.

### 4.2. Questionnaire Development

In this study, we propose a theoretical framework on the basis of the previous literature and social learning and social exchange theory. In this framework, despotic leadership strengthens employee job satisfaction directly and indirectly through self-efficacy and leader–member exchange. First, we develop the research questionnaire in English, and then translate it into the Chinese language. We translated the questionnaire into Chinese because the respondents of this study were Chinese speakers. After translating the questionnaire, we conducted a pilot study of the questionnaire to measure its reliability and validity. The pilot study participants were 15 employees of SMEs, 5 Ph.D. students, and 5 university professors. The participants of the pilot study recommended some changes to the questionnaire, and we revised the instrument as per the pilot study participants’ recommendations. Later, we distributed the questionnaire among the employees of small- and medium-sized enterprises (SMEs) located in Guangdong Province (China).

### 4.3. Variables Measurements

Despotic leadership is the independent variable of this study. The scale was designed by De Hoogh and Den Hartog [19]. Moreover, the scale is based on the motivated strategies for learning questionnaire (MCLQ), and consists of three items, which are “my supervisor expects subordinates to absolutely obey”, “my supervisor is bossy, and he or she acts like a tyrant”, and “my supervisor likes to give orders and does not tolerate disagreement or questioning him or her” (Cronbach’s alpha = 0.749).

As the dependent variable in this work, job satisfaction is measured using the Minnesota satisfaction scale, which was designed by Weiss [61] and repeatedly tested by numerous studies, and is highly authoritative in academia. Considering that too many items were found in the original scale, this study selected a condensed version with four items, including “I can get along well with my colleagues in the company”, “I am satisfied with the attitude of my boss”, “I can get a sense of achievement from my work”, and “I am satisfied with the current working environment of the company” (Cronbach’s alpha = 0.751).

Self-efficacy is the mediating variable of this study. The scale in this paper consists of three items with a high load from the general self-efficacy scale compiled by Schwarzer and Bäßler [62]. Three items are contained in the scale, which are “if I try my best, I can always solve problems in my work”, “I am confident that I can deal effectively with any unexpected situation at work”, and “even if others object to me, I can still achieve my goal” (Cronbach’s alpha = 0.779).

Leader–member exchange is the moderating variable in this study, for which the three-item scale developed by Scandura and Graen [63] was adopted. The three items of LMX included “my immediate supervisor is the kind of person who makes people willing to make friends with”, “I admire the knowledge and ability of my supervisor in his work”, and “I like the person of my direct supervisor very much” (Cronbach’s alpha = 0.711). In this study, we considered two control variables: age and position, because age and position connect with self-efficacy and employees’ job satisfaction.

### 4.4. Sample and Data Collection

The target population was workers working in SMEs located in Guangdong Province, China. In the questionnaire, the authors informed the respondents that the information they were providing would be confidential, and that the information would be used only for research purposes. We conducted an online survey through the www.wjx.cn website. This website is a third party that provides services to collect data from the target population. We distributed 500 questionnaires among the employees of SMEs in the vicinity of Guangdong Province, P.R. China. We received 265 research questionnaires; 35 were discarded due to non-pertinent or atypical cases. The final sample consisted of 230 responses, which is 53% of the distributed questionnaires.

### 4.5. Demographics

Table 1 presents the demographic characteristics of the samples in this work. In terms of the sex ratio of the respondents, 42.61% of the respondents were female, and 57.39% were male, which reflects the gender balance of the sample. In terms of age, the majority of the respondents were over 40 years old. Specifically, 40.43% were 41–50 years old, and 40.87% were 50 years old or above. Moreover, 6.96% of the samples were aged 30 or below, and 11.74% of the respondents were 31–40 years old. From the perspective of educational background, 4.78% had graduate degrees or higher, 33.48% had bachelor’s degrees, 37.83% had college degrees, and 11.3% had finished high school or below. From the perspective of the respondents’ position, most were managers, accounting for 72.6% of the respondents; 18.7% were senior management; and 8.7% were ordinary staff.

## 5. Analysis and Results

We used AMOS-27 software (IBM, New York, USA) for analysis of the findings and their reliability, confirmatory factor analysis, and regression analysis. We conducted confirmatory factor analysis (CFA) on the empirical model, and tested the factor composition of each variable and the fitting effect of the model. It provided support for the evaluation of the construct validity and the reliability of the collected samples. Moreover, for the hypotheses confirmation, we employed covariance-based SEM. In this research, the two main reasons for why we adopted covariance-based SEM instead of partial least squares structural equation modeling (PLS-SEM) are summarized. The first reason is that the structural model is complex and involved mediating relations [64,65]. The second reason is that we exploited covariance-based SEM due to its less stringent conditions for preventive hypotheses, which encourages investigators to draw and evaluate such models by aiding them in avoiding extra preventive constraints [66]. In addition, particularly for the measurement of the descriptive statistics of the variables, the inter-variable correlation, and the ANOVA test, we used SPSS-25 (Statistical Package for the Social Sciences) (IBM, New York, NY, USA). It helped us to understand the general tendencies of the constructs, their association among the variables, and the difference tendencies of the control variables for given variables.

### 5.1. Reliability and Validity

Prior to data analysis, the reliability and validity of all the study constructs were vigilantly checked. The reliability for all the measurement scales was analyzed through Cronbach’s alpha, which is a common tool for checking the reliability of constructs. The standard acceptable value of Cronbach’s alpha is more than 0.70. Table 2 presents the alpha values of each construct as greater than 0.70, which indicates the reliability of all the variables. Moreover, Tompson and Barclay [67] and Hair and Ringle [68] suggested that the standard acceptable values of composite reliability (CR) and rho_A should be higher than 0.70. Table 2 presents the CR and rho_A values of each construct as greater than 0.70. Finally, average variance extracted (AVE) was used to measure the convergent validity of the constructs. It was observed that all AVE values crossed the threshold of 0.5 [69], as given in Table 2.

#### 5.1.1. Discriminant Validity

Discriminant validity of the construct provides empirical evidence about the constructs and how much the constructs differ from each other. It also provides the difference level among overlapping constructs. It can be measured with the criterion of Fornell and Larcker [69]. This method compares the latent constructs’ correlation with the square root of the average variance extracted (AVE) values of each construct. The latent construct should explain the variance of its relevant indicators instead of the variance of other constructs. The square root of all constructs should have a higher correlation with them as compared to other constructs [69], as given in Table 3.

### 5.1.2. Model Fitness

The model in this paper has shown a good fitting degree, and the validity of the scale was ideal according to the suggested model fit indicators by Hu and Bentler [70]. Table 4 indicates that the value of X^2^/df is 1.157, and is less than the standard value of 3. The RMSEA value is 0.026, and is under the standard value of 0.05. The GFI value is 0.990, and is close to the standard value of 0.9. The AGFI value is 0.932, and is also close to the standard value of 0.9. The CFI, IFI, and TLI values are over 0.950, and are much greater than the standard value of 0.9. To sum up, the results of confirmatory factor analysis confirm that the empirical model composed of despotic leadership, job satisfaction, self-efficacy, and LMX has good validity; the collected data have high reliability, and the research results have strong robustness. The overall fitting degree of the empirical model is shown in Table 4.

### 5.2. Descriptive Statistics and Correlation

To test the correlation between each variable, this research used the Pearson correlation coefficient analysis method through SPSS 25 to conduct a correlation analysis on the sample data of the returned questionnaire and to obtain the correlation coefficient between the two variables. The results confirmed that the four variables included in the empirical model were significantly correlated. Among them, despotic leadership was significantly negatively correlated with job satisfaction, leader–member exchange, and self-efficacy, while job satisfaction is significantly positively correlated with leader–member exchange and self-efficacy. Moreover, LMX and self-efficacy are significantly positively correlated with each other. Table 5 shows the correlation between the core and the control variables.

#### Control Group Differences

Control variables of different position groups and age groups have shown a moderately positive and significant correlation with the variables self-efficacy (SE) and Job Satisfaction (JS). ANOVA was applied to measure the difference in self-efficacy and job satisfaction for different position groups such as general staff, supervisors, managers, and senior management. There was a difference between different employees’ job positions for their self-efficacy (F = 45.68, *p* < 0.001), as well as their job satisfaction (F = 51.48, *p* < 0.001). The post hoc Tukey HSD test was run to measure the differences between the different positions and self-efficacy, as well as job satisfaction. It was observed that grass-roots staff had a higher level of self-efficacy and job satisfaction as compared to middle-level managers. Middle-level managers had higher SE and JS than top-level senior management.

Moreover, in this study, the authors applied ANOVA to measure the differences in self-efficacy and job satisfaction for different age groups. There was a difference between different age groups (less than 30 years old, 31–40 years old, 41–49 years old, and 50 years old or above) for their self-efficacy (F = 47.27, *p* < 0.001) as well as for their job satisfaction (F = 48.06, *p* < 0.001). The post hoc Tukey HSD test was run to measure the differences between the different age groups (less than 30 years old, 31–40 years old, 41–49 years old, and 50 years old or above) for self-efficacy and job satisfaction. It was observed that the 30 years of age or less group had a higher level of self-efficacy and job satisfaction than the 30–40 years of age group. Similarly, the 30–40 years of age group had higher SE and JS than the 41–49 years of age group. The 41–49 years of age group had a higher level of SE and JS than the 50 years of age or above group.

### 5.3. Hypothesis Testing

In this study, for the hypotheses testing, we employed structural equation modeling to measure the direct, indirect, moderation, and mediated moderation effects. Hayes’ [71] process model 7 was applied to measure the mediated moderation with AMOS. The DL was considered as an independent construct, while LMX was placed as the moderator, SE was the mediator construct, and JS was the dependent variable. An interaction term “DL_LM” was also created through the multiplication of DL and LMX. Position and age were considered as control variables. The minimum model fit criteria [70] was achieved for the results of the direct and indirect effects, moderation, and mediated moderation.

#### 5.3.1. Direct Effects

First, the direct effects of the association of DL with JS were measured. The results confirmed that despotic leadership has a direct and negative significant relationship with job satisfaction (β = −0.343, SE = 0.06, *p* < 0.001). Thus, H1 was accepted as given in Table 6.

#### 5.3.2. Indirect Effects

To measure the mediation effect of SE between DL and JS, a two-step procedure by Preacher et al. [72] was followed. First, the significant relationship between the independent and mediating variables (X→M) was measured, and then, the relationship between the dependent variable and the mediating variable (M→Y) was measured. Both conditions were met; therefore, we proceeded to measure the mediating effect of SE between DL and JS. The results showed that the indirect effect of LMX on JS through SE was positive and significant (**β** = 0.129, SE = 0.033, *p* < 0.001). Thus, H2 was not rejected, as shown in Table 7.

#### 5.3.3. Moderation Effects

**Figure 2:**
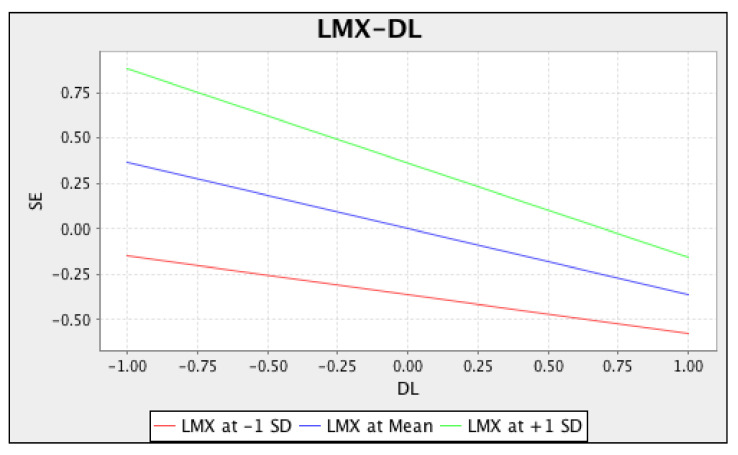
Moderating effect of LMX on SE with the independent variable DL.

After measuring the direct and indirect effect, the moderating effect of LMX was analyzed. The results showed that the interaction term (DL X LMX) had a significant negative effect on SE (β = −0.105, SE = 0.054, *p* < 0.05). To understand the nature of the significant interaction of the effect of DL and LMX, a graph of the interaction effect was drawn as given in Figure 2. The graph represented a negative relationship between DL and SE, which was observed to be weaker when LMX was observed high as compared to when it was low. Figure 2 presents the moderating effect of LMX on SE with the independent variable DL.

#### 5.3.4. Mediated Moderation

Following the validation of H1 and H2, the mediated moderation of the LMX, which represented a significant moderating effect, was analyzed. Following Epitropaki [73], the estimands were defined in AMOS as an alternate of the PROCESS macro for SPSS [74] to measure the mediated moderation by utilizing a model in which the moderator has influence at the first stage (X→M) of the mediation association (X→M→Y). The level, bootstrapped at 2000, resulted from three values of LMX (−1 SD, mean SD, and +1 SD), supporting the conditional indirect effect (CIE) of DL on JS, which reduced with the values of the moderator, as given in Table 8.

**Figure 3:**
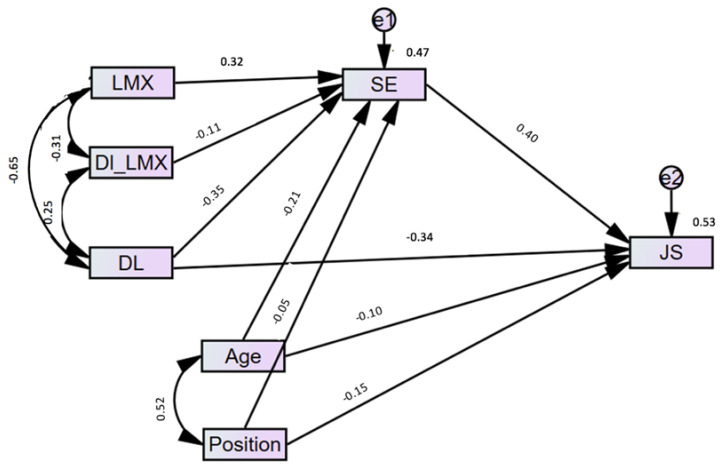
Structural equation modeling.

More precisely, the values of the negative indirect effect of DL on JS through the mediation of SE was significant at high LMX at all three levels, as given in Table 8. The result supported hypothesis three (H3). The overall direct relationships among the variables are shown in Figure 3; similarly, the overall summary of the hypotheses is given in Table 9.

## 6. Discussion

Prior studies on leadership have discussed the bright side of leadership style. In this study, we have discussed the dark side of leadership, which shows the impact of despotic leadership on job satisfaction. Despotic leadership is a negative leadership style that affects employees’ job performance. Moreover, this study also expressed the mediating role of self-efficacy between the relationship of despotic leadership and job satisfaction. Similarly, the leader–member exchange is moderating in the relationship between despotic leadership and self-efficacy.

First, we focused on the direct relationship between despotic leadership and employee job satisfaction. The results showed that despotic leadership has a negative impact on employees’ job satisfaction, which supported H1. The findings of this study were also supported by the previous studies [75,76]. Similarly, Ofori [77] conducted a study on the relationship between despotic leadership and employees’ outcomes, the finding of his study indicated that despotic leadership negatively affects employees’ job satisfaction. This study was also supported by the social learning and social exchange theory [78]. However, in order to maintain the image of leaders and display the advantages of power, despotic leaders often show a strict and autocratic side to their employees, which makes employees feel unhappy, which, in turn, affects their performance [24]. Kasi and Bibi [79] demonstrated that a despotic leader will destroy the fair atmosphere of an organization, and they tend to promote those individuals who are ingratiatory, rather than appointing individuals according to their ability [8]. Therefore, the above studies indicated that despotic leaders negatively affect the job satisfaction among employees, which supports our findings.

Second, the findings of this study confirmed that self-efficacy plays a mediating role between despotic leadership and employee job satisfaction. Therefore, the outcomes are consistent with previous studies and support H2. This result is also in line with the social learning theory [80]. Naseer and Raja [5] indicate that despotic leaders were not encouraging towards their subordinates, in addition to such kind of leaders being used to punishing their employees according to their mood. Therefore, it is difficult for the employees to show their talents and gain a sense of self-efficacy from their previous experiences. However, despotic leadership negatively affects self-efficacy in results, and affects the employee’s job satisfaction [81].

Third, we tested the moderating effect of LMX between despotic leadership and self-efficacy. The findings of this study demonstrated that LMX moderates between despotic leadership and self-efficacy, which supports H3. Therefore, the results of this study also confirm that LMX reduces the negative impact of despotic leadership on employees’ self-efficacy. These results are in line with the theory of social exchange and the research of De Clercq and Fatima [8]. The hypothesized relationship-moderating effects between despotic leadership and self-efficacy are based on leader–member exchange theory [82], which supports our findings. Moreover, with the high quality of LMX, the insider will continue to perceive the leader’s recognition and tolerance for their uniqueness in their work. In return for these gifts from the leaders, they tend to be more diligent and positive in their work, and thus have a stronger sense of self-efficacy. On the contrary, in the case of low-quality LMX, outsiders tend to obtain fewer resources and opportunities, and it is difficult for them to get recognition, even for their achievements. As a result, their self-confidence and motivation for progress are, inevitably, gradually lost, and their sense of self-efficacy is lowered [52].

In this study, we considered two control variables, age and position. The t-test and ANOVA were tested to measure whether an employee’s position influences their self-efficacy and job satisfaction. The findings of this study confirmed that grassroots staff had a higher level of self-efficacy and job satisfaction compared to middle-level managers, and middle-level managers had higher SE and JS than top-level senior management. Moreover, in this study, the authors applied ANOVA to measure the differences in self-efficacy and job satisfaction for different age groups. The outcomes of this study confirmed that the 30 years of age or less group had a higher level of self-efficacy and job satisfaction than the 30–40 years of age group. Similarly, the 30–40 years of age group had higher SE and JS than the 41–49 years of age group, and the 41–49 years of age group had a higher level of SE and JS than the 50 years of age or above group.

## 7. Conclusions

The outcomes of this study show the relationships between despotic leadership, leader–member exchange, self-efficacy, and job satisfaction. Specifically, despotic leadership has a direct negative impact on job satisfaction, while self-efficacy acts as a mediating role between despotic leadership and job satisfaction. Similarly, the leader–member exchange is moderating in the relationship between despotic leadership and self-efficacy.

The conclusions of this paper are as follows: first, despotic leadership will have a harmful effect on the majority of employees in the organization, and it will be difficult for employees to achieve ideal job satisfaction under such a leader. Secondly, despotic leadership damages employees’ job satisfaction through the reduction of their self-efficacy. Despotic leaders are often overconfident and autocratic, which makes them less able to encourage or empower employees. Therefore, employees cannot gain self-efficacy from successful experience and the recognition others, and are thus less able to get satisfaction from their work. Finally, when encountering despotic leaders, employees can establish appropriate personal relationships with the leader to reduce the negative impact of despotic leadership on their self-efficacy and job satisfaction.

## 8. Contributions, Limitations, and Future Research

### 8.1. Theoretical Contribution

First, from the employees’ perspective, this work deepens the negative impact of despotic leadership. Given the limitation of data availability, early research on leadership mainly focused on the positive impact of leadership on enterprises and employees, and investigations on negative leadership styles are rare [8]. As a destructive leadership style prevalent in Eastern cultures, despotic leadership has long had a wide impact on the employees of SMEs, but only in recent years has it gradually attracted the attention of researchers [5]. At present, the existing literature primarily concentrates on the overall impact of despotic leadership on the organization, and rarely goes deep into the micro-level of employees to explore the micro-impact of this leadership style on employees’ psychology and work experience [19,83]. Based on the social learning theory by Walker and Morley [80], this study proposes an empirical model whereby despotic leadership reduces job satisfaction by influencing employee self-efficacy. According to the test results, despotic leadership’s strong controlling desire will lead to “learned helplessness” from the employees, a circumstance that will cause the loss of their self-efficacy and, ultimately, the perceived significance of their work. These findings broaden the academic understanding of the mechanism of despotic leadership and enrich the research context of social learning theory.

Second, from the perspective of social exchange, this work explores the coping mechanisms of the negative effects of despotic leadership. In the previous literature, few studies have examined the direct relationship between despotic leadership and job satisfaction or between despotic leadership and leader–member exchange, but the four-way relationship between despotic leadership, leader–member exchange, self-efficacy, and job satisfaction has been, for the first time, explored in this study. In particular, this study considered self-efficacy as an intervening construct and leader–member exchange as a moderating construct between despotic leadership and job satisfaction. According to the empirical test results, we posit that LMX can enhance the trust and emotion of leaders toward employees to provide more empowerment and support to the latter. The empowerment and support of leaders can better promote employee self-efficacy. Thus, the leader–member exchange can weaken the negative impact of despotic leadership on employee self-efficacy. These findings provide strategic guidance for employees to deal with despotic leadership and further confirm the practical value of social exchange theory.

### 8.2. Practical Implications

First, the employees of SMEs should acknowledge the negative effects of despotic leadership and take personality and leadership style as important reference standards in the recruitment and selection of managers so as to maximally prevent leaders with a strong despotic style from joining the management. Second, enterprises must formulate strict and fair rules and regulations to help employees establish correct work values to prevent an unhealthy atmosphere of pursuing individual authority and autocracy within the enterprise, and prohibit the emergence of “small groups” in an effort to improve employee job satisfaction and work efficiency. Finally, from the employees’ perspective, they should take the initiative to maintain a good relationship with despotic leaders, should they appear. According to the theory of social exchange, “insiders” are more likely to be valued by despotic leaders, thereby gaining richer resources and opportunities. Even if they make mistakes, “insiders” are more likely to be forgiven and tolerated by leaders and have higher satisfaction with their jobs. Regardless of the leadership style, employees should pay attention to maintaining a certain connection with the leader and strive to maintain the authority of the leader in the process of work. Of course, if employees find that they do not get along with their supervisor, they should “flee” as soon as possible to prevent loss.

### 8.3. Limitations and Future Research

This study has some limitations, which should be considered when interpreting the results. This study was cross-sectional, and as such, does not provide inference on causality. Like many prior studies, the study used a self-reporting technique, which may be subjected to social desirability bias. Secondly, the findings of this study only investigated small- and medium-sized enterprises. Further research can be undertaken in the IT, education, or manufacturing sectors. It generalized the outcomes and modified the concepts. Thirdly, given the lack of research resources and limited availability, this study only examined the issue from the perspective of subordinates, and did not conduct a matching survey on subordinates and their leaders. Moreover, this work used a cross-sectional approach, and only focused on the psychological state of employees at a given time, a circumstance that may lead to the problem of common method deviation. Although the data analysis found no serious common method deviation, future research will benefit from collecting data from multiple time points for comparison and reference and dynamically examining employees’ work experience.

## Figures and Tables

**Table 1:** Demographics of the respondents.

Measure	Items	Frequency	Percentage (%)
Gender	Male	132	57.39
	Female	98	42.61
Age	30 or below	16	6.96
	31–40	27	11.74
	41–49	93	40.43
	50 or above	94	40.87
Education background	Senior high school or below	55	23.91
	Junior college	87	37.83
	Bachelor’s degree	77	33.48
	Postgraduate or above	11	4.78
Position	Grass-roots staff	20	8.70
	Middle manager	167	72.60
	Top manager	43	18.70

**Table 2:** Reliability and validity of the construct.

Constructs	Item Loading	Alpha	rho_A	CR	AVE
**Despotic Leadership**		0.749	0.749	0.810	0.588
DL1	0.766				
DL2	0.743				
DL3	0.791				
**Job Satisfaction**		0.751	0.751	0.843	0.572
JS1	0.765				
JS2	0.743				
JS3	0.747				
JS4	0.771				
**Leader–Member Exchange**		0.711	0.712	0.794	0.562
LMX1	0.741				
LMX2	0.740				
LMX3	0.768				
**Self-Efficacy**		0.779	0.779	0.824	0.610
SE1	0.743				
SE2	0.808				
SE3	0.791				

**Note:** Alpha, Cronbach’s alpha; CR, composite reliability; AVE, average variance extracted; DL, despotic leadership; JS, job satisfaction; LMX, leader–member exchange; SE, Self-efficacy.

**Table 3:** Fornell–Larcker Criterion.

No	Constructs	1	2	3	4
1	Despotic leadership	0.767			
2	Job satisfaction	−0.677	0.757		
3	LMX	−0.651	0.654	0.75	
4	Self-efficacy	−0.642	0.713	0.651	0.781

**Note:** LMX = leader–member exchange.

**Table 4:** The fitting degree of the empirical model in this paper.

X^2^/df	RMSEA	GFI	AGFI	CFI	IFI	TLI	*p*
1.157	0.026	0.956	0.932	0.990	0.991	0.987	0.192

**Note:** RMSEA, root mean square error of approximation; GFI, goodness-of-fit index; AGFI, Adjusted Goodness-of-Fit Index; CFI, comparative fit index; TLI, Tucker–Lewis index.

**Table 5:** Descriptive statistics and construct correlation.

No	Constructs	M	SD	1	2	3	4	5	6	7	8
1	Despotic Leadership	3.48	0.80	1							
2	LMX	2.50	0.77	−0.653 **	1						
3	Self-Efficacy	2.53	0.83	−0.642 **	0.650 **	1					
4	Job Satisfaction	2.58	0.80	−0.675 **	0.656 **	0.713 **	1				
5	Gender	1.43	0.50	0.018	−0.092	−0.067	−0.025	1			
6	Age	3.15	0.89	0.449 **	−0.524 **	−0.558 **	−0.520 **	0.031	1		
7	Education	3.08	1.05	0.234 **	−0.180 **	−0.186 **	−0.174 **	0.02	−0.055	1	
8	Position	2.73	0.86	0.502 **	−0.443 **	−0.473 **	−0.528 **	0.01	0.515 **	−0.03	1

**Note:** ** *p* < 0.01 (two tails); * *p* < 0.05 (two tails); M, mean; SD, standard deviation; LMX, leader–member exchange.

**Table 6:** Direct effects.

Construct			*β*	S.E.	*p*-Value	R^2^
Self-Efficacy	←	Age	−0.211	0.066	***	0.474
Self-Efficacy	←	Position	−0.051	0.060	0.358	
Self-Efficacy	←	LMX	0.318	0.064	***	
Self-Efficacy	←	Despotic Leadership	−0.347	0.058	***	
Self-Efficacy	←	Despotic Leadership_LMX	−0.105	0.054	0.037	
Job Satisfaction	←	Age	−0.096	0.060	0.079	0.528
Job Satisfaction	←	Position	−0.154	0.061	0.004	
Job Satisfaction	←	Self-Efficacy	0.404	0.057	***	
Job Satisfaction	←	Despotic Leadership	−0.343	0.060	***	

**Note:** *** *p* < 0.001); LMX = leader–member exchange.

**Table 7:** Standardized Indirect Effects.

Construct					*β*	SE	*p*-Value
Job Satisfaction	←	Self-Efficacy	←	Position	−0.021	0.025	0.072
Job Satisfaction	←	Self-Efficacy	←	Age	−0.085 **	0.028	***
Job Satisfaction	←	Self-Efficacy	←	Despotic Leadership _LMX	−0.043 *	0.032	0.041
Job Satisfaction	←	Self-Efficacy	←	Despotic Leadership	−0.140 **	0.032	***
Job Satisfaction	←	Self-Efficacy	←	LMX	0.129 **	0.033	***

**Note:** *** *p* < 0.001); LMX = leader–member exchange.

**Table 8:** Direct and Indirect Mediating Moderating effect.

Parameter	B	Lower		Upper	*p*
lowSS	−0.241		−0.378	−0.088	0.001
medSS	−0.332		−0.442	−0.221	0.001
highSS	−0.423		−0.597	−0.282	0.001
lowCIE	−0.093		−0.161	−0.033	0.002
medCIE	−0.128		−0.192	−0.075	0.001
highCIE	−0.162		−0.253	−0.096	0.001
IndModMed	−0.045		−0.105	−0.001	0.044

**Table 9:** Summary of the results of hypothesis testing in this paper.

Hypothesis	Test Results
**H1.** *Despotic leadership has a negative impact on employee job satisfaction.*	Supported
**H2.** *Self-efficacy mediating between despotic leadership and employee job satisfaction.*	Supported
**H3.** *Leader-member exchange moderate between despotic leadership and employee self-efficacy.*	Supported

## Data Availability

The data will be available on request.
